# Neointimal coverage of bare-metal and sirolimus-eluting stents evaluated with optical coherence tomography

**DOI:** 10.1136/hrt.2007.118679

**Published:** 2007-10-08

**Authors:** B X Chen, F Y Ma, W Luo, J H Ruan, W L Xie, X Z Zhao, S H Sun, X M Guo, F Wang, T Tian, X W Chu

**Affiliations:** Department of Cardiology, Beijing Electric Power Hospital, Beijing, China

## Abstract

**Objective:**

To analyse the neointimal coverage of sirolimus-eluting stent (SES) and bare-metal stent (BMS) visualised in vivo by optical coherence tomography (OCT).

**Methods:**

OCT images were obtained in 26 coronary vessels of 24 patients at 5–93 months after SES or BMS deployment. The short-term BMS group (BMS1) consisted of eight BMS in seven patients at 5–10 months of follow-up, the long-term BMS group (BMS2) consisted of six BMS in six patients at 23–93 months of follow-up, and the SES group (SES) consisted of 13 SES in 10 patients at 6–12 months of follow-up. The strut apposition, strut coverage and mean maximal and minimal neointimal thicknesses (NIT) for both BMS groups and SES were compared.

**Results:**

OCT images were acquired successfully. Significant differences between completely apposed and malapposed stent struts (p<0.0001) and between covered and uncovered stent struts (p<0.0001) were found among the three groups. The mean maximal and minimal NIT in the SES group were all significantly less than those of the BMS1 or BMS2 group, the minimal NIT in the BMS1 group was significantly less than that of the BMS2 but the mean maximal NIT was no significant difference between the BMS1 and BMS2 groups. In an open bifurcation artery, 19 struts visualised by OCT had no discernible coverage or were surrounded by either thrombus or a thick tissue layer.

**Conclusions:**

OCT imaging can clearly visualise stent apposition and neointimal coverage of stent struts. Incomplete strut apposition and lack of strut coverage occurred with a significantly higher frequency in SES than in BMS. These findings may explain the occurrence of late thrombosis in SES.

Optical coherence tomography (OCT) is the optical analogue to ultrasound, measuring the back-reflection of infrared light instead of sound waves. The greatest advantage of OCT is its high resolution, which exceeds that of any currently available in vivo imaging technology. The resolution of catheter-based systems is in the range of 10–20 μm.^1^ ^2^ Furthermore, resolutions as high as 4 μm have been achieved ex vivo with more sophisticated techniques that may be applicable to future catheter-based approaches. The main components of various atheromatous plaques can be identified in OCT images, and have been validated in a histology-controlled study.^3^ Several studies have demonstrated the feasibility of OCT imaging in patients undergoing percutaneous coronary intervention (PCI).^4^^–^10 The aim of the present study was to use OCT to analyse the neointimal coverage of sirolimus-eluting stents (SES), compared with that of bare-metal stents (BMS).

## METHODS

### Study population

From July 2005 to November 2006, 24 patients (21 men and 3 women) at 5–93 months of follow-up after SES or BMS implantation in a native coronary artery were enrolled in the study after written informed consent was obtained. Patients were excluded if they had left main disease, ostial disease, congestive heart failure or renal insufficiency with baseline serum creatinine >1.8 mg/dl (>133 μmol/l). Coronary angiograms were obtained from all patients before OCT imaging. OCT images of 28 stents in 26 diseased coronary vessels were obtained from the 24 patients. The treated vessel segments were distributed as follows: 18 left anterior descending (LAD) lesions, treated with 19 stents (68%); four left circumflex (LCX) lesions, treated with five stents (18%); and four right coronary artery (RCA) lesions, treated with four stents (14%). An equal number of BMS and SES (n = 14; eight Cypher, Cordis Johnson & Johnson and six Firebird, Microport) were implanted among the study population. The short-term BMS group (BMS1) consisted of eight BMS in seven patients at 5–10 months follow-up. The long-term BMS group (BMS2) consisted of six BMS in six patients at 23–93 months of follow-up and the SES group (SES), consisted of 13 SES in 10 patients at 6–12 months of follow-up. Antiplatelet therapy consisted of clopidogrel for one month and aspirin indefinitely for both BMS groups, and clopidogrel for 12 months and aspririn indefinitely for the SES group. One patient who experienced acute anterior myocardial infarction 29 months after SES stenting in the LAD was excluded from the SES group.

### OCT image acquisition

After coronary angiography, a 0.014 inch (0.36 mm) guidewire was advanced through a 6Fr guiding catheter into the desired vessel and across the target location, and then a 4F Helios occlusion balloon catheter (OBC, LightLab Imaging, Westford, MA, USA) was advanced over the guidewire into the vessel until the OBC tip marker was distal to the target location. The guidewire was removed from the OBC, and the 1.4F ImageWire (LightLab Imaging, Westford, MA, USA) was advanced to the OBC distal tip marker under fluoroscopic guidance. While holding the ImageWire in place, the OBC was retracted until the balloon marker was located proximal to the target site. The contrast injection pump was turned on to start the flow of flush solution at 0.5–0.6 ml/s through the OBC lumen. The occlusion balloon was then inflated to 0.3–0.5 atm. When an OCT image of the vessel wall appeared, a motorised pullback was initiated from the imaging system console. At a pullback rate of 1.0 mm/s (or 1.5 mm/s) and 15 frames/s, a video sequence of cross-sectional OCT images over a 3 cm (or 4.5 cm) length of artery was obtained. After imaging was complete, the balloon was deflated, and flow of flush solution was stopped.

### OCT data analysis

OCT images were analysed by two independent investigators who were blinded to the clinical presentation. When there was discordance between the observers, a non-blinded consensus reading was completed. One OCT cross-sectional still frame was selected for quantitative analysis from each 1 mm segment throughout the entire length of each stent. The still frames were selected based on appearance of stent struts, and lack of OCT image artefacts such as motion artefacts. Each observed strut in each analysed still frame was classified as either completely or incompletely apposed, with a distance of ⩾200 μm between the strut inner-surface reflection and vessel wall defined to be incomplete apposition. The surface of every strut was also classified as either covered or uncovered. For each covered strut, the maximal and minimal neointimal thicknesses (NIT) were measured and these measurements were compiled to obtain the mean maximal and minimal NIT for each stent. Finally, the strut apposition, strut coverage and mean maximal and minimal NIT for BMS and SES were compared.

### Statistical analysis

Continuous data are expressed as mean (SD). Baseline characteristics and measurements were analysed by use of the Pearson χ^2^ test or one-way ANOVA, as appropriate. All analyses were performed using a statistical software package (SPSS version 11.5). A p value of ⩽0.05 was specified as the confidence level for statistical significance.

All imaging procedures were performed without complication or adverse events.

## RESULTS

### Baseline characteristics

In the short-term BMS group (BMS1), the average time between the stenting and OCT imaging follow-up was 7.29 (SD 2.06; range 5–10) months, versus 44.50 (24.56; 23–93) months for the long-term BMS group (BMS2) and 8.60 (2.72; 6–12) months for the SES group. No significant differences were found in the demographic or baseline characteristics, including coronary risk factors, between the groups (table 1).The implanted stent diameters and lengths among three groups were also not significantly different.

**Table 1:** Baseline characteristics

Characteristic	BMS1	BMS2	SES	p Value
Cases	7	6	10	
Age (years)	61.0 (10.49)	64.2 (7.78)	58.3 (9.82)	0.50
Time post-stenting (months)	7.29 (2.06)	44.50 (24.56)	8.60 (2.72)	<0.0001*
Male sex	6	6	8	0.51
Stent diameter (mm)	3.03 (0.34)	3.04 (0.25)	3.05 (0.28)	0.99
Stent length (mm)	18.75 (3.77)	17.50 (3.99)	22.00 (6.03)	0.15
Hypertension	5	6	8	0.38
Diabetes mellitus	1	2	6	0.16
Smoking	3	5	6	0.33
CHO (mmol/l)	4.33 (0.88)	4.03 (0.36)	4.51 (1.13)	0.50
TG (mmol/l)	1.37 (0.52)	2.08 (1.03)	2.00 (1.53)	0.77
HDL (mmol/l)	0.91 (0.43)	1.04 (0.09)	1.03 (0.22)	0.80
LDL (mmol/l)	2.81 (0.69)	2.35 (0.22)	2.88 (0.97)	0.39
OCT target vessel				
LAD	4	4	9	0.24
LCX	1	0	3	
RCA	2	2	0	

BMS1 group, bare-metal stent with 5–10 months follow-up; BMS2 group, bare-metal stent with 23–93 months follow-up; SES group, sirloimus-eluting stent with 6–12 months of follow-up; cholesterol (CHO); triglyceride (TG); high-density lipoprotein cholesterol (HDL); low-density lipoprotein cholesterol (LDL); OCT, optical coherence tomography; left anterior descending artery (LAD); left circumflex artery (LCX); right coronary artery (RCA).*Within three groups, p<0.0001; BMS1 vs SES, p = NS.

### OCT imaging

The OCT findings are summarised in table 2.

**Table 2:** OCT findings

Finding	BMS1 (n = 7)	BMS2 (n = 6)	SES (n = 10)	p Value
No of stents	8	6	13	
Total stent struts	1465	1242	2425	
Side branch struts	3	7	24	
Complete apposition	1462	1235	2358	<0.0001*
Incomplete apposition	0	0	43	
Strut covered	1457	1231	1990	<0.0001**
Strut uncovered	5	4	368	
Maximal NIT (mm)	0.59 (0.36)	0.61 (0.27)	0.12 (0.07)	<0.0001†
Minimal NIT (mm)	0.20 (0.18)	0.22 (0.15)	0.04 (0.02)	<0.0001‡

* SES vs BMS1 and BMS2, p <0.0001;**SES vs BMS1 and BMS2, p<0.0001; †within three groups, p<0.0001, BMS1 vs BMS2, p = NS; ‡within three groups, p<0.0001, BMS1 vs BMS2, p = 0.028.

**Figure 1:**
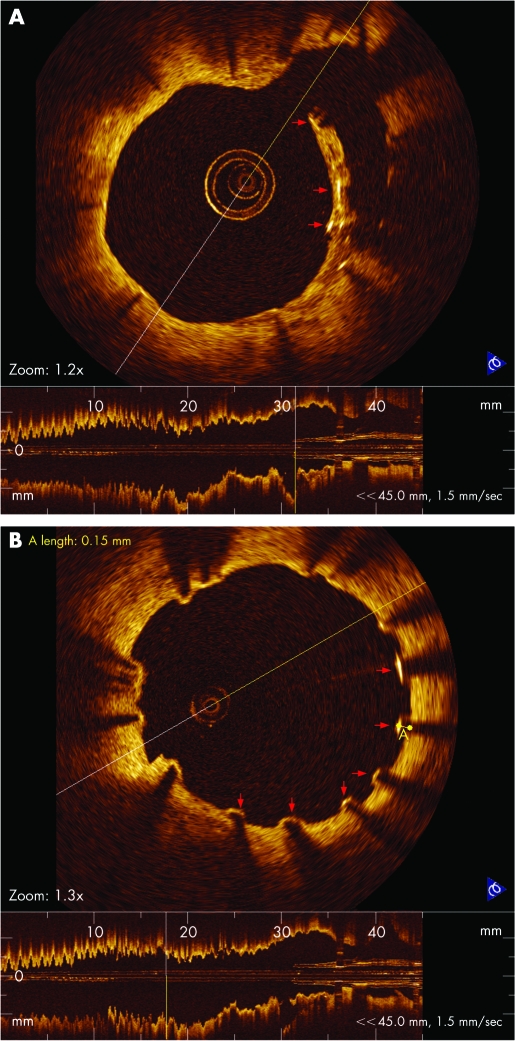
(A) OCT image of stent struts surrounded by a thick tissue that fuses with vessel wall in an open bifurcation artery in SES 12-month follow-up (red arrow). (B) OCT imaging visualised of uncovered struts (red arrow) in SES 12-month follow-up.

**Figure 2:**
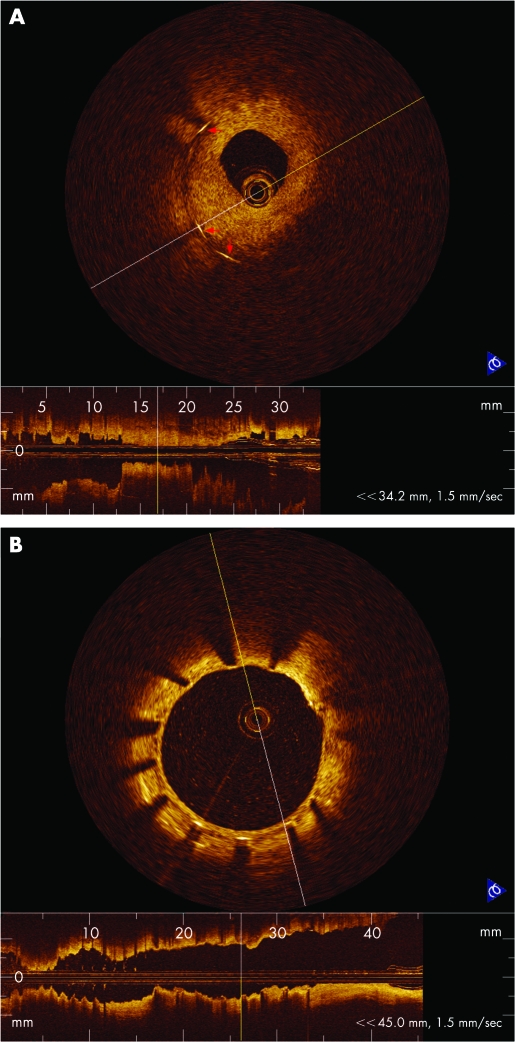
(A) OCT image showing struts surrounded by hyperproliferative neointimal growth which led to re-stenosis 5 months after BMS stenting. (B) OCT image of well apposed stent struts with a thin neointimal formation around stent 12 months after SES stenting.

The total number of stent struts evaluated was 1465 struts in the BMS1 group, 1242 in the BMS2 group and 2425 in the SES group. Of all the stent struts, three stent struts within the open bifurcation artery were completely surrounded by thrombus in the BMS1 group; six struts were surrounded by thrombus and one strut was surrounded by a thin tissue fusing with vessel wall in the BMS2 group; and 24 struts observed within the open bifurcation artery in the SES group showed complex conditions, including a few struts surrounded by thrombus, others surrounded by a thick tissue layer (fig 1A), and the remaining almost uncovered. Significant differences among the percentages of completely apposed stent struts (100%, 100% and 98%, in the BMS1, BMS2 and SES groups, respectively; BMS1 vs SES p<0.0001; BMS2 vs SES p<0.0001) and covered stent struts (99.7%, 99.7% and 83%, in the BMS1, BMS2 and SES groups, respectively; BMS1 vs SES p<0.0001; BMS2 vs SES p<0.0001) (figs 1, 2A, 2B) were found among the three groups, but there was no significant difference between the BMS1 and BMS2 groups. The mean maximal and minimal NIT measured in the SES group were all significantly less than that of BMS1 or BMS2 (p<0.0001), the minimal NIT in the BMS1 group was significantly less than the BMS2, but mean maximal NIT was no significant difference between the BMS1 and BMS2.

**Figure 3:**
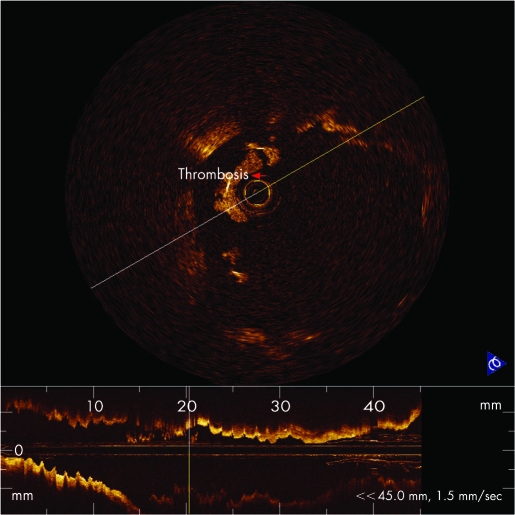
OCT image clearly showed strut surrounded by thrombus (red arrow).

One patient experienced acute anterior myocardial infarction (AMI) after SES stenting (Cypher) in the LAD at 29-month follow-up. Fifteen days after AMI, coronary angiography showed no significant stenosis in LAD with TIMI grade 3 flow. OCT examination of the LAD showed irregular stent strut separation, stent malapposition, thrombus surrounding the surfaces of stent struts and a dilated coronary artery within the stented segment (fig 3).

## DISCUSSION

With the development of percutaneous coronary intervention (PCI), intracoronary stenting has become the treatment of choice for patients with focal coronary artery disease. Until about five years ago, bare-metal stents were applied widely in PCI, but a major problem with re-stenosis resulting from hyperproliferative neointimal growth became apparent in a significant proportion of patients 3–6 months after stenting, especially for patients with high coronary risk factors. In the era of drug-eluting stents such as sirolimus-eluting stents, re-stenosis rates have been reduced dramatically for high-risk patients, but one of the major concerns is the biocompatibility of these stent systems. Evidence from recent studies suggests that the anti-proliferative effects of SES delay re-endothelialisation within the stent, increasing the risk of stent thrombosis.^11^^–^13 Late or very late thrombosis after SES stenting is related to lack of the stent strut endothelial coverage. In order to understand and control the in vivo processes that take place after SES implantation, additional new tools are needed beyond angiographic guidance.

OCT is a catheter-based technology producing images from backscattered “echoes” similar to intravascular ultrasound (IVUS), but using a near-infrared, high-bandwidth light source. This new technology provides ultra-high resolution (10 μm) for in vivo endovascular visualisation. Ex vivo studies have shown resolution capabilities of 2–10 μm, which provide the necessary definition to identify the key elements of vulnerable plaques, including the thin fibrous cap, lipid-rich core and high macrophage content.^3^ ^7^ ^14^ ^15^ This new imaging method is also capable of detecting several different features of intravascular pathology, such as previous disruption of fibrous caps of lipid-rich plaques, the presence of the intracoronary thrombosis, the depth of dissections caused by balloon inflations that can not be fully appreciated by IVUS, cuts in the atherosclerotic plaque made by the blades of a cutting balloon, tissue protruding through stent struts and underdeployed struts otherwise missed by IVUS. Several studies have demonstrated the feasibility of intravascular OCT for obtaining qualitative information for characterisation of coronary atherosclerotic plaques and for evaluation of intracoronary stenting.^5^ ^6^ ^8^ ^9^

In the present study, we employed OCT to analyse strut apposition and neointimal coverage of BMS and SES at follow-up in 24 patients with coronary artery disease. In BMS, OCT images showed all stent struts with complete apposition, with almost all strut surfaces covered by neointima, as well as some struts covered by hyperproliferative neointima, which resulted in re-stenosis. In the 5–10-month and 23–93-month follow-up groups, the strut apposition, strut coverage, and maximal NIT were not significantly different. These results indicate that strut apposition and neointimal thickness were stable in BMS 5 months after stenting. Compared with BMS, SES visualised by OCT had more incomplete apposition, with more struts uncovered and thinner NIT. These results verify the results of numerous earlier studies that show SES can significantly reduce neointimal growth and re-stenosis in the short-term follow-up. However, more SES stent struts were uncovered or malapposed compared to BMS stent struts. Both of these conditions may lead to late or very late thrombosis.

One patient in our study experienced an acute anterior myocardial infarction 29 months after the implantation of a sirolimus-eluting stent. This patient had been imaged using OCT 14 days after AMI. Although coronary angiography did not find any evidence of restenosis or thrombosis, intracoronary OCT revealed irregular strut separation and fibrin-rich thrombus. The precise mechanisms related to the stent malapposition and late thrombosis in this case are unknown. A recent report from Virmani *et al* describes the first case of fatal acute myocardial infarction and cardiac rupture resulting from late thrombosis of a Cypher stent deployed 18 months previously.^16^ The autopsy in the case reported by Virmani *et al* showed an aneurysm in the wall of the stented artery, with extensive inflammatory infiltration consisting of lymphocytes, plasma cells, macrophages and eosinophils invading the intima, media and adventitia. The luminal surface of the proximal stent was surrounded by fibrin-rich thrombus with sparse smooth muscle cells, and the luminal surfaces of the proximal and distal stents were focally malapposed, with thick layers of fibrin thrombus separating the stent from the underlying plaque and arterial wall. The authors concluded that localised hypersensitivity vasculitis developed in response to the polymer of the Cypher coronary stent was the most likely mechanism. The OCT imaging results of our AMI patient were similar to the findings of the autopsy reported by Virmani *et al*.^16^

From these findings of OCT imaging in SES or BMS, we conclude that long-term strut malapposition and the lack of neointimal coverage of stent struts may be responsible for late or very late thrombosis, and that enhanced surveillance of patients with SES may help avoid some of the observed late complications with SES. Moreover, continued efforts to improve polymer biocompatibility in SES are warranted.

### Limitations

OCT imaging has some limitations in clinical situations, one of which is its inability to image through blood. Therefore, it is necessary to establish a blood-free environment for OCT light to penetrate to the tissue. Although clear OCT imaging was obtained by the use of OBC balloon blocking blood flow, the need to occlude blood limits the clinical application of OCT, especially for patients with left main disease, ostial disease, congestive heart failure and very tortuous lesions. Another limitation is the limited depth of penetration of OCT, which is 1.5–2.0 mm. The 10 μm resolution of OCT means that stents with 10 μm of very early re-endothelial coverage may have unknowingly been categorised as uncovered in this study. The second generation OCT imaging system may eliminate many of the technical limitations of the present study.
